# Plasticity of adipose tissues in response to fasting and refeeding declines with aging in mice

**DOI:** 10.18632/aging.204734

**Published:** 2023-05-23

**Authors:** Ya-Ru Chen, Fen Xiao, Hao-Neng Tang, Ting Wang, Ying-Hui Zhou, Junaid Iqbal, Shui-Bing Yang, Long Li, Houde Zhou

**Affiliations:** 1 National Clinical Research Center for Metabolic Diseases, Hunan Provincial Key Laboratory for Metabolic Bone Diseases, and Department of Metabolism and Endocrinology, The Second Xiangya Hospital of Central South University, Changsha 410011, Hunan, China; 2 Department of Laboratory Medicine, The Second Xiangya Hospital, Central South University, Changsha 410011, Hunan, China; 3 Department of Endocrinology, The First People's Hospital of Huaihua, Huaihua 418000, Hunan, China

**Keywords:** age, fasting and refeeding, adipose tissue, fat mobilization, plasticity

## Abstract

To explore the plasticity of adipose tissues, C57BL/6J mice at the age of 1 month, 3 months, and 15 months corresponding to adolescence, adulthood, and middle-aged transitional period, respectively, were fasted and refed subsequently at different times. Body adipose tissues ratio (BATR) was calculated, the morphology of adipose tissue and the area of adipocytes were observed by histological analysis, and the mitochondria in adipocytes were observed under the transmission electron microscope. Furthermore, the expression levels of *Ucp-1*, *Cidea*, *Cox7a1*, *Cpt-1m*, *Atgl*, and *Hsl* were detected by qRT-PCR. Our results showed a significant increase in the adipocytes area and body visceral adipose tissue (VAT) ratio in all groups of mice with aging. Moreover, body mesenteric white adipose tissue (mWAT) ratio decreased the most after 72 h fasting. In the middle-aged transitional mice, the white adipocytes did not decrease until 72 h fasting, and most of them still appeared as unaffected unilocular cells. Besides, the number of mitochondria and the expression of *Ucp-1*, *Cidea*, *Cox7a1*, *Cpt-1m*, *Atgl* and *Hsl* were lower in these mice. After 72h refeeding, the body subcutaneous white adipose tissue (sWAT) ratio returned to normal, while the VAT kept decreasing. The above results indicated an impairment in adipose tissue plasticity in mice with aging, suggesting that age modulated the metabolic adaptiveness of adipose tissues in mice.

## INTRODUCTION

Obesity is a major risk factor for many metabolic diseases and affects the outcome of long-term metabolic abnormalities in patients [1, 2]. The incidence of obesity peaks at the transitional period of middle-aged and elderly individuals. With age, the energy storage function of adipose tissue declines, resulting in hyperlipidemia and fat redistribution [3]. Aging is characterized by complex changes in a variety of biological functions of the body over time, including the imbalance of energy metabolism. Multiple age-related genetic changes affect the body’s energy metabolism and adipose tissues play a vital role in maintaining energy homeostasis [4].

The size and distribution of fat dramatically change with age. WAT gradually expands, and the swelling of adipose tissue correlates with its size. Adipocyte volume is the smallest during adolescence, reaches the peak in the transition period of middle-aged or early old age followed by a sharp decline [5]. Hypertrophic fat cells have lower insulin sensitivity, increased lipolytic capacity, elevated free fatty acid (FFA) and cholesterol levels, causing hyperlipidemia and metabolic dysfunction of the entire system [6, 7]. The main function of brown adipose tissue (BAT) is to produce heat and increase energy expenditure. There is evidence that the number and function of BAT gradually decrease with age, and so does the beige fat [8]. Hence, many studies investigated BAT to prevent and treat obesity [9–11].

Adipocytes can break down or store fat through instant structural and functional changes to maintain energy homeostasis. This phenomenon is known as the plasticity of adipose tissue. Adipose tissue exhibits varying extent of plasticity under different environmental conditions to maintain energy homeostasis [12]. (1) In response to cold, individual adipocytes remodel their internal architecture to facilitate thermogenesis, causing browning. Secondly, cold induces the production of new adipocytes from adipogenic progenitor cells via de novo differentiation. (2) White adipocytes can reversibly dedifferentiate *in vivo* and *in vitro,* such as during lactation (dedifferentiation), involution (redifferentiation), and hair-follicle cycling. (3) Adipose tissue has a remarkable ability to expand and contract. Expansion is mediated by two mechanisms: hypertrophy (increases in individual adipocyte size) and hyperplasia (increases fat-cell number mediated by de novo differentiation of adipocyte progenitor cells). During fasting, adipocytes release nutrients into the systemic circulation by breaking down stored triacylglycerols (TAGs) and releasing free fatty acids (FFAs) and gradually shrink and become smaller. These changes are reversible and adipocyte phenotype is restored with the removal of the biological/physiological stimuli. However, the loss of adipose tissue plasticity leads to a decline in adipose tissue function during obesity and aging [13]. It is believed that the adipose tissue expands beyond the reach of blood vascular supply, causing tissue hypoxia and accumulation of senescent cells. Fibrosis and inflammation of adipose tissue eventually led to insulin resistance and metabolic diseases.

An in-depth understanding of the comprehensive effects of aging and nutritional interventions, such as calorie restriction and fasting and refeeding, on adipose tissue is very important for preventing and treating obesity and metabolic disorders. According to epidemiological surveys, the prevalence of obesity is the highest among middle-aged and elderly individuals in the transitional age, who usually also suffer from insulin-resistant type 2 diabetes [14, 15]. Despite the middle-aged transitional period being a turning point between adulthood and old age, many age-related diseases often manifest during the middle-aged transitional period [16]. Majority of the studies have focused on young (3-6 months old) and old (20-24 months old) C57BL/6J mice. The middle-aged transitional period (12-18 months old) is often ignored in research. Our previous study showed that fasting preferentially consumed VAT, and fat droplets were mobilized in large numbers under fasting, which suggested the malleable nature of adipose tissue during fasting and refeeding [17]. Fasting and refeeding can challenge energy metabolism and influence adipocytes’ basic cellular and molecular functions, making age-related changes in adipose tissue plasticity more pronounced. Therefore, in this study, we compared the morphology, histology and gene expression changes of various adipose tissues in different age groups, especially the middle-aged transitional male C57BL/6J mice after fasting and refeeding, in order to prevent obesity and promote weight loss during the transition period.

## RESULTS

### The effects of aging on body weight and adiposity of male mice

**Figure 1:**
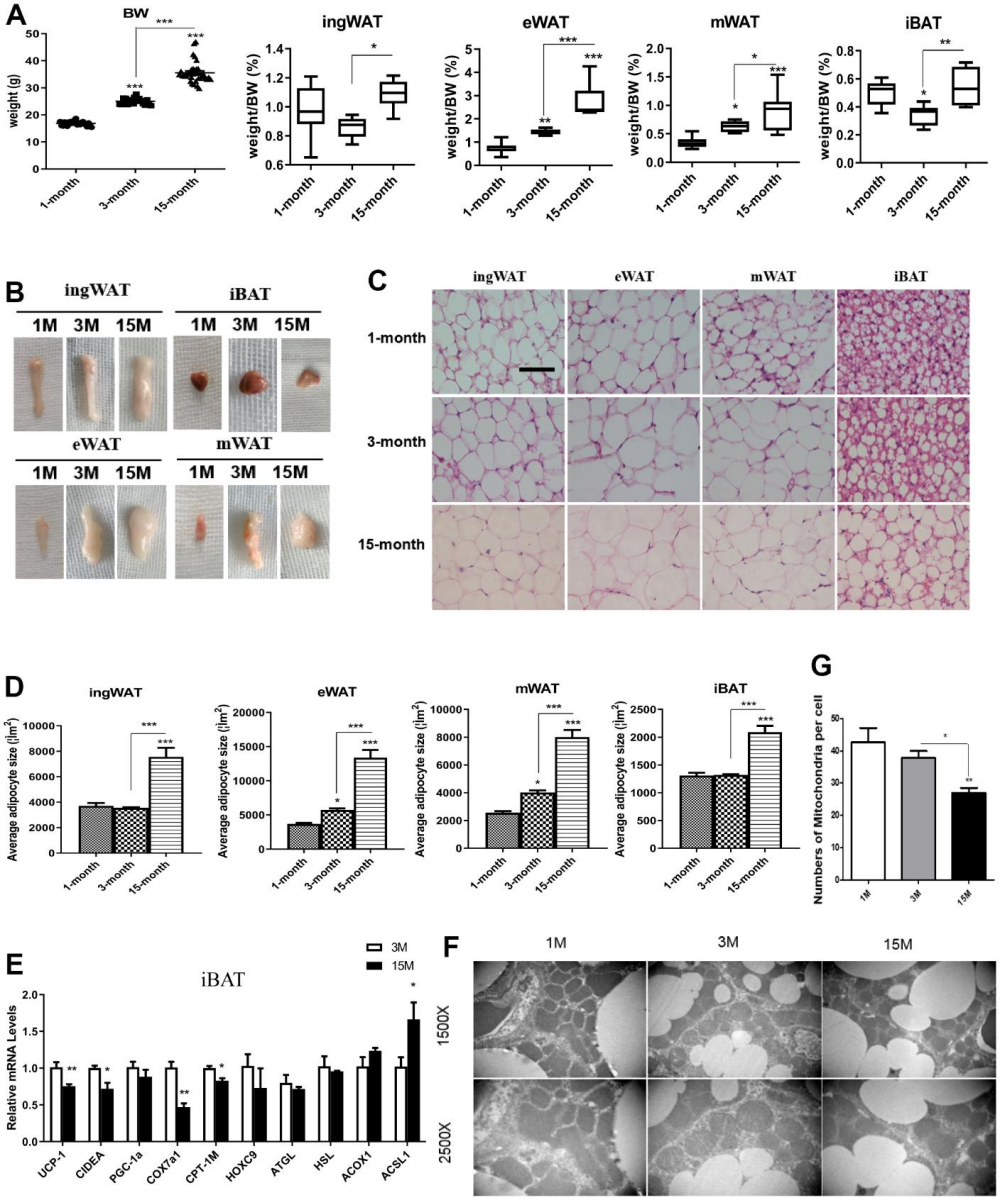
**Differences in fat mass and fat distribution between young, adult and middle-age transitional mice.** Young mice (1-month-old), adult mice (3-month-old) and middle-age transitional mice (15-month-old) were fed ad libitum. Body weight and body adipose tissues ratio of inguinal white adipose tissue (ingWAT), epididymal WAT (eWAT), mesenteric WAT (mWAT), interscapular BAT (iBAT) of mice (**A**) and their appearance (**B**) were analyzed (n = 6–12). The effect of aging on histological characteristics of white and brown adipose tissues (**C**). The average area of adipocytes (μm^2^) in every 100-mm^2^ area range of various adipose tissues was quantified using the Image Pro Plus software (**D**) (n = 6-8). Scale bar represents 100 μm. Differential expression of mitochondrial biogenesis related gene (*Pgc-1α*) and thermogenic gene (*Ucp-1*) (**E**) were measured (n = 6). Representative transmission electron microscopy (TEM) images of brown adipose tissues from the three groups of mice fed ad libitum and mitochondrial numbers (**F**, **G**) were analyzed. All data are presented as the mean ± SEM. * *p* < 0.05; ** *p* < 0.01; *** *p* < 0.001.

As shown in Figure 1A, the bodyweight of mice of different age groups was different. Among the three groups, the bodyweight of middle-aged transitional mice group was the highest. We measured adipose tissue weight from the four different depots of ad libitum (AL)-fed mice against the total body weight, and found the weight of the fat depots in late-middle aged mice to be the highest (Figure 1B). There was a noticeable increase in both the size and percent weight of visceral fat, particularly epididymal WAT (eWAT) with increasing age. Compared with the adolescent group, the weight of the inguinal WAT (ingWAT) and interscapular BAT (iBAT) from adult mice were both decreased slightly, but the proportion of ingWAT and iBAT in the middle-aged transitional mice were higher than that in the other groups. The proportion of ingWAT (1%) was the highest among all the other types of adipose tissues in adolescent mice, while the proportion of eWAT increased with age, from 0.71% in the adolescent group to 2.75% in middle-aged transitional group.

To determine whether the expanded fat mass resulted from an increase in cell number or cell size, we performed histological analysis on the four types of adipose tissues from all the different groups of mice (Figure 1C, 1D). Morphological examination showed that the area of adipose cells increased with age, which was consistent with the appearance of adipose tissue. The size of adipocytes was largest in the middle-aged transitional mice in all fat depots, while there was no significant change in adipocyte size in adult mice relative to the adolescent group. Adipocytes became larger during the period of adult to middle-age transition, and the area of white adipocytes and brown adipocytes doubled. In addition, a small number of multilocular adipocytes were observed in the WAT of young mice, showing a distinct “brown-like” morphology. The percentage of multilocular adipocytes decreased with age and they disappeared in the middle-aged transition group. On the contrary, the increase of unilocular adipocytes (white-like morphology) was more obvious in the middle-aged transitional group.

Loss of multilocular adipocytes during the aging process indicated an impairment of the thermogenic ability of iBAT. Therefore, we conducted a PCR analysis to examine the impact of morphological changes on brown adipocyte–specific gene expression (Figure 1E). We found that the expression of *Ucp1* was markedly reduced in 15-month mice relative to younger animals. Expression of other thermogenesis and lipolysis-related genes, such as *Cidea* and *Cpt1m*, and mitochondrial biogenesis genes, such as *Cox7a-1* were also significantly reduced. To further understand the “white-like” brown adipocytes, TEM was used to assess the ultrastructure of cells (Figure 1F, 1G). The results showed a significant decrease in the number of mitochondria between middle-age transitional group and adult group, and no significant reduction between the young and adult group.

### The impact of fasting and refeeding on body weight and adiposity of mice at different ages

**Figure 2:**
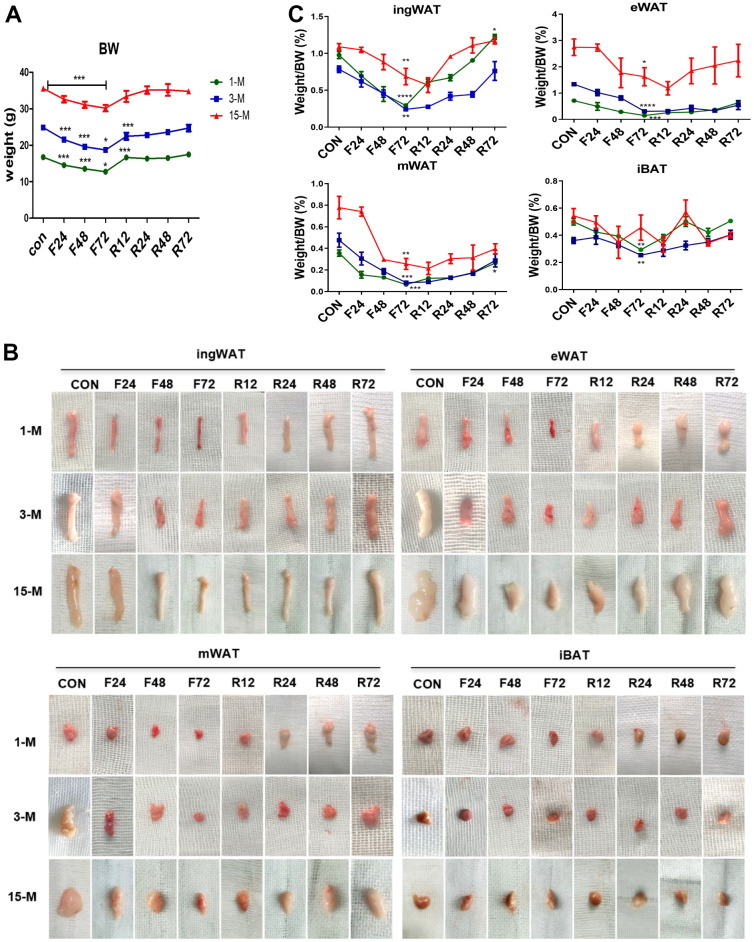
**Change in the body weight and fat mass of mice belonging to the different experimental groups subjected to fasting and refeeding.** All mice were fasted for 24, 48 and 72 h (F24 h, F48 h and F72 h) respectively, and then fed again for 12, 24, 48 and 72 h (R12 h, R24 h, R48 h and R72 h), respectively. After 72 h of fasting, the body weight and morphology of various adipose tissues (**A**, **B**) from the different age groups were evaluated. Weights of adipose tissues (ingWAT, eWAT, mWAT, iBAT) expressed as a percentage of body weight in mice of the three groups (**C**) were also analyzed. All data are presented as the mean ± SEM.* *p* < 0.05; ** *p* < 0.01; *** *p* < 0.001 compared with control mice (CON).

To investigate the impact of aging on the capacity for coping with energy deprivation, we fasted mice of different age for 24 h, 48 h, and 72 h (F24 h, F48 h, and F72 h), and subsequently refeeding for 12 h, 24 h, 48 h, and 72 h (R12 h, R24 h, R48 h, and R72 h). At first, we evaluated the effect of fasting and refeeding on the body weight of the three age groups (Figure 2A). Fasting markedly reduced their body weights, and especially after F72 h, it decreased the body mass of the young, adult, and the middle-age transitional mice by 23.4%, 24.6%, and 15.1%, respectively (*p* < 0.05). However, after 3 days of refeeding the lost body mass of the young and adult group was restored or even higher than the original weight (increased by 4.4% and 1.2% respectively), while a continuous weight loss of 4.9% was observed in the middle-aged transitional group. It is worth noting that the speed of weight loss and regain was higher in both young and adult groups than in the middle-aged transitional group.

Four adipose depots were collected from all the animals at each time point (Figure 2B). Compared to the control group, F72 h fasting promoted a greater loss of BATR in the young (83.11%), adult (75.66%) and the middle-aged transitional mice (40.91%), respectively. Moreover, compared to the control group, after R72 h, the total body white adipose tissues ratio (BWATR) of the three groups was significantly reduced by 29.86%, 38.74% and 11.63%, respectively.

To investigate the possible depot-specific responses in fasted mice, we compared the changes in the amount of each white fat depot. We found that the body fat ratios of all WAT depots were significantly reduced after F72 h. Compared with their normal feeding control group, the body fat ratio of ingWAT after 72 h fasting was lower by 69.89%, 69.18%, and 36.98% in young, adult and middle-age transitional mice, respectively; eWAT decreased by 78.69%, 59.38%, and 34.41% in young, adult and middle-age transitional mice, respectively; mWAT decreased by 81.88%, 82.64%, and 67.08% in young, adult and middle-age transitional mice, respectively. Among the four types of adipose tissue, mWAT showed the greatest decrease in body fat percentage (Figure 2C). Among three groups of mice, we found that the body adipose tissue ratio of all four fat depots of the middle-aged transitional mice decreased the least. After R72 h, ingWAT ratio was restored or even higher than the initial ingWAT (by 1.25% in young mice), while VAT (eWAT, and mWAT) continued to be low in all groups of mice. During fasting, the rate of reduction in each white fat depot in young and adult mice was steady, while in the middle-aged transitional group, it was the slowest in the first F24 h, followed by an abrupt acceleration in the second F24 h, after which it was back to a slower loss in the last F24 h. To our surprise, we found that the tendency for decrease in each white fat depot still continued after R12 h in the middle-age transitional group, while the percentage of WAT of the young and the adult began to increase. Fat restoration began after R 24 h in the middle-age transitional group.

### Change in adipocytes and lipolytic enzymes in mice of different ages during fasting and refeeding

**Figure 3:**
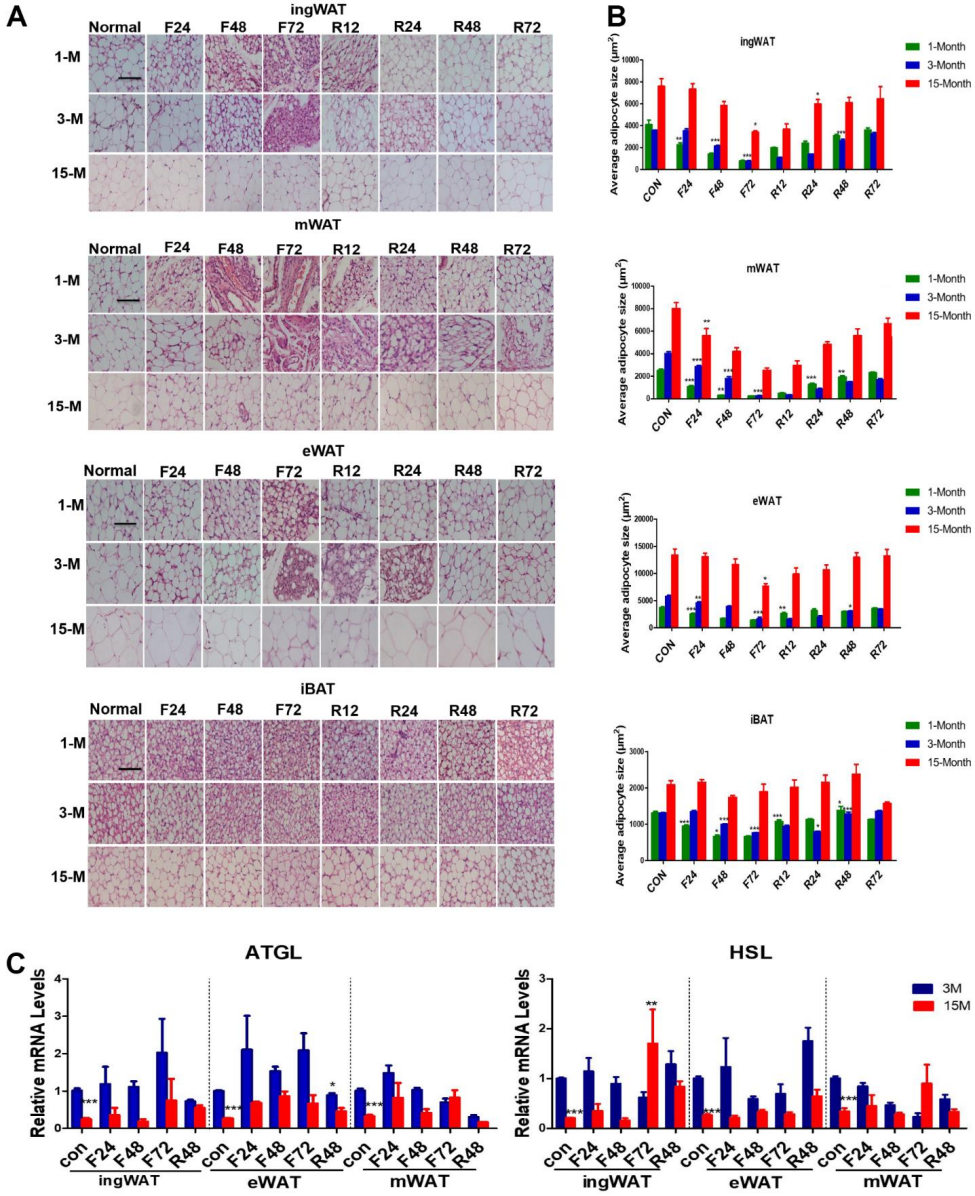
**Histological alterations of adipose tissue in the three groups of mice subjected to fasting and refeeding.** The effects of fasting and refeeding on histological alterations of various adipose tissues in adult mice (**A**). The average area of adipocytes (μm^2^) in every 100-mm^2^ area range of various adipose tissues was quantified using the Image Pro Plus software (**B**) (n = 6-8). Expression of lipid metabolism-related genes in different white fat depots in adult and middle-age transitional mice under different feeding conditions. Differential expressions of lipid mobilization related genes (*Atgl, Hsl*) (**C**) in inguinal, epididymal and mesenteric adipose tissues were measured (n = 6–8). Scale bar represents 100 μm and all data are presented as the mean ± SEM. * *p* < 0.05; ** *p* < 0.01; *** *p* < 0.001 compared with control.

We next tested whether fasting also influenced the morphology of different types of adipose tissues (Figure 3A, 3B). White adipocytes rapidly mobilize a considerable amount of intracellular stored fat to release non-esterified fatty acids (NEFA) providing energy during food deprivation. White adipocytes and the stored intracellular fat decreased in size during fasting, which could be easily visualized by histological examination. Therefore, microscopic evaluation of the WAT tissue sections from young and adult groups subjected to F24 h showed a considerable reduction in lipid droplet and adipocyte size. In contrast, F24 h failed to significantly reduce adipocyte and lipid droplet size in the WAT of middle-age transitional mice until after F72 h. Unilocular fat cells mixed with multilocular fat cells were observed in the WAT of young and adult mice, which did not appear in middle-aged transitional mice until being fasted for 120 h (not shown). Some unaffected unilocular cells were observed in the ingWAT and eWAT even after F72 h. At the same time, there were completely delipidated adipocytes in the mWAT already after F24 h and hardly any lipid droplets were present after F48 h in young and adult mice, which indicated that the stored fat in mWAT was the first to mobilize.

Analyzing the average area of adipocytes (Figure 3B) also showed that adipocyte size of mWAT from the three groups was the first to decrease after F24 h (by 50% in young mice, 25% in adult mice, and 25% in the middle-age transitional mice), whereas that of eWAT and sWAT remained unchanged or modestly reduced after F48 h. After F72 h, adipocyte size in mWAT decreased by 99%, 99%, and 60%, in the young, adult and middle-age transitional mice, respectively. The eWAT was reduced by 60%, 75%, and 20%, and the ingWAT by 80%, 85%, and 30% in young, adult and middle-age transitional mice, respectively.

After refeeding, adipocytes from the three depots in the different age groups showed expansion. The rate of restoration was the fastest in young mice, where ingWAT increased by 100%, eWAT by 50%, and mWAT by 50% after R12 h. In contrast, there was no significant change in adult and middle-age transitional mice. By R72 h, all adipocytes from the three depots in young mice recovered to the level of the fed group, while in adult and middle-age transitional mice, they were still slightly smaller than the fed group.

Given the different fat mobilization rates of adipocytes from different age group mice, we explored the effects of aging on the expression of the two major lipolysis related enzymes, *Atgl* (adipose triglyceride lipase, Atgl) and *Hsl* (hormone-sensitive lipase, Hsl), when exposed to negative energy balance (Figure 3C). As we expected, the mRNA expression levels varied depending on the adipose tissue age, depot, and the duration of fasting. Under normal feeding conditions, the expression of *Atgl* and *Hsl* in the different fat depots of the middle-aged transitional mice were lower than those of adult mice (*p* < 0.05). After fasting, the expression level of *Atgl* was higher in all the fat depots of adult mice. After R48 h, the gene expression decreased in adult mice, but the expression of *Atgl* in the middle-aged transitional mice did not change significantly.

### The effects of fasting on blood serum parameters at different ages

**Figure 4:**
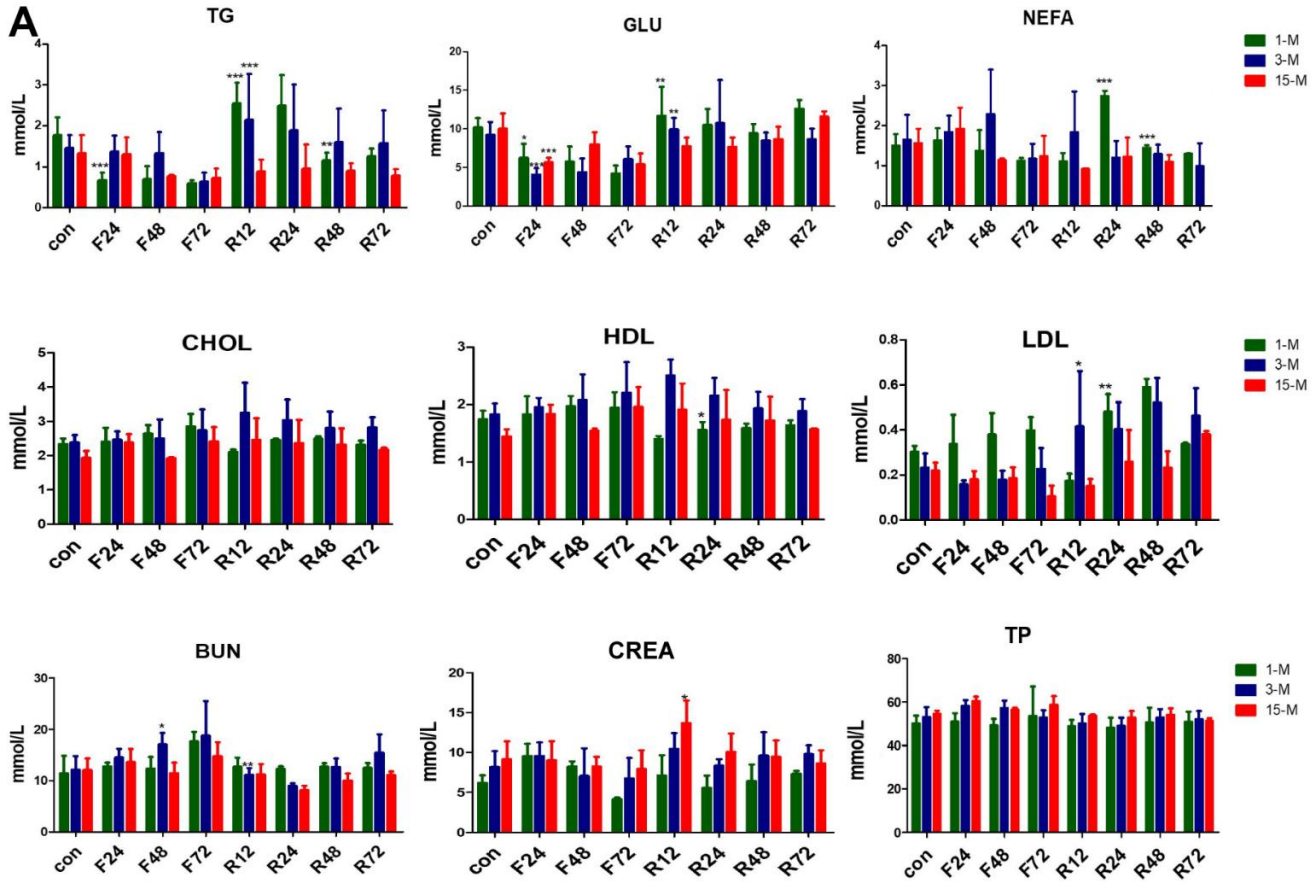
**Serum concentrations of metabolites in the three groups of mice subjected to fasting and refeeding.** (**A**) All data are presented as the mean ± SEM. * *p* < 0.05; ** *p* < 0.01; *** *p* <0.001.

Basal serum concentrations of TG, FFA, total cholesterol, and HDL cholesterol etc. are shown in Figure 4. GLU, TG, NEFA, and ALB showed no significant difference among the three age groups. The young and adult groups showed similar results except for LDL and CREA, whereas CHOL, HDL, LDL were reduced in the middle-aged transitional group, LDL level deceased with age, and CREA level was higher in the old age group.

After F24 h, the serum levels of GLU and TG were significantly reduced in all the three groups of mice. Upon 72 h fasting, this change became insignificant. After feeding for another 12 hours, the serum levels of GLU and TG of the adolescent and adult groups rose rapidly. Although there was a slight upward trend in the middle-age transitional group, it was not statistically significant. The NEFA levels of all the three groups of mice tended to increase after 24 h fasting. The NEFA levels of the adult group continued to increase until fasting for 48 h. Subsequently, the NEFA of the three groups of mice decreased. After refeeding, the NEFA levels of the adolescent and adult groups increased rapidly, but that of the middle-age transitional mice did not.

## DISCUSSION

The plasticity of adipose tissue is influenced by many factors such as age, diet, and external stimulus such as temperature. It accumulates or releases stored lipids by changing its structure and function [17, 18]. The plasticity of WAT is specifically altered upon excess energy consumption, which is achieved through (1) adipocyte hypertrophy and/or recruitment and proliferation of pre-adipocytes (2) recruitment of inflammatory cells (3) blood vessels and extracellular matrix remodeling, to ensure WAT expands enough to store excess energy. The plasticity of BAT is characterized by the enhancement of BAT function caused by energy intake, increase in heat production, and energy consumption [19]. However, the functional decline of adipose tissue during obesity and aging is associated with a loss of plasticity. Aging is inevitable and associated with several metabolic ailments, including obesity. Aging-induced defects in adipocyte progenitor cells (APCs) include decreased expression of sirtuins, reduced expression of pro-adipogenic transcription factors, and impaired proliferative capacity [20]. Secondly, the abundance and activity of thermogenic adipose tissue decreases in aging [21]. The conversion of low-thermogenic cells to high-thermogenic cells in BAT gets impaired with aging [22]. Moreover, aging in mice also leads to the accumulation of proinflammatory aging-dependent regulatory cells (ARCs). ARCs impede proliferation and adipogenic capacity of APCs, and thus presumably impair de novo beige adipogenesis with age [23]. In addition, immune cells play critical roles in regulating adipose-tissue phenotypes in response to physiological and pathological stimuli. As reported in regulatory T-cells (Treg), the level of ILC2 was reduces in adipose-tissue in the setting of obesity. There is also a decrease in the abundance of ILC2 expressing and these cells also lose their identity in the visceral adipose tissue of mice during aging [24]. Therefore, taking advantage of the enormous plasticity of adipose tissue is a very promising approach to treat age-related metabolic diseases.

**Figure 5:**
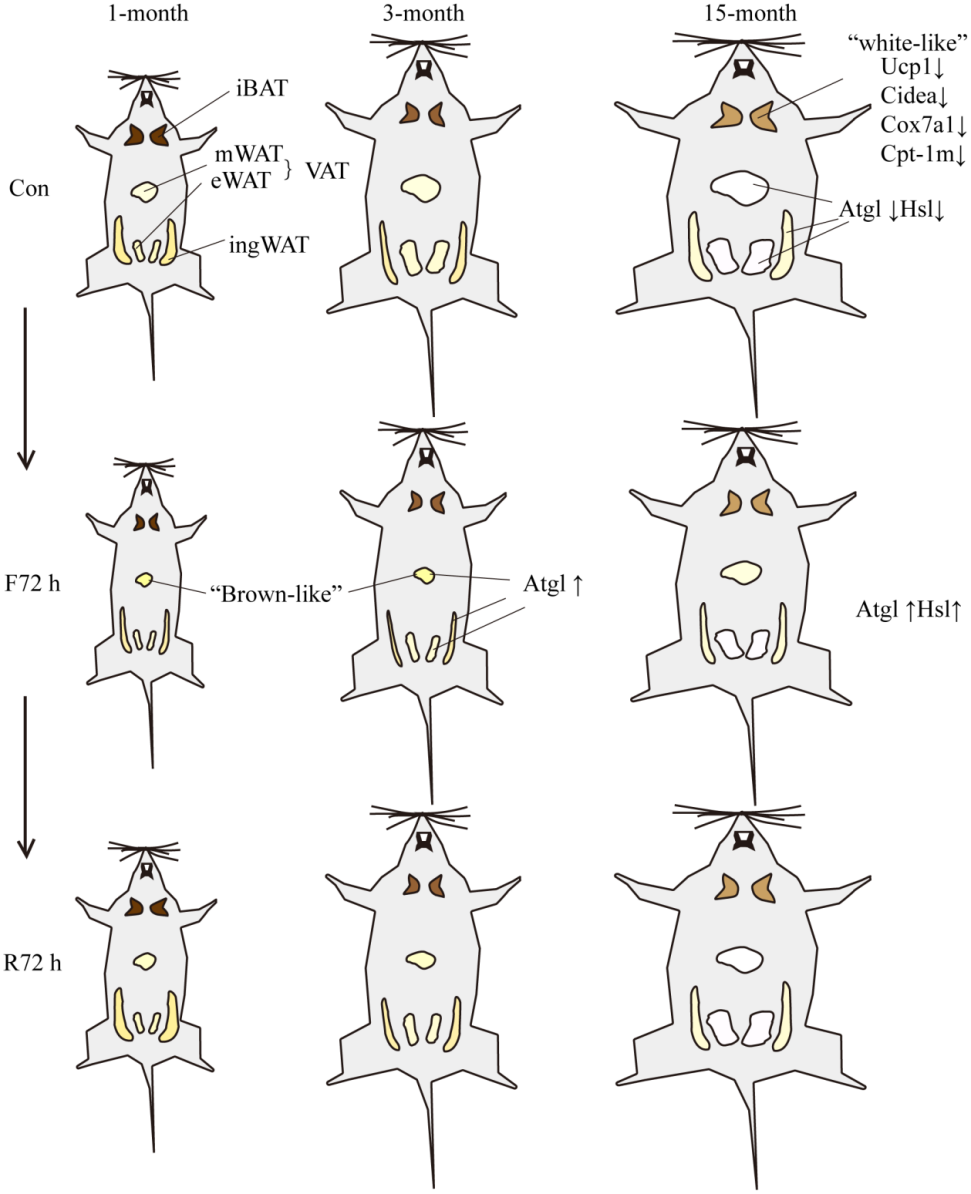
**Schematic showing the response of mice of different ages to fasting and refeeding.** With the increase of age, the body weight and BATR of all four fat depots increased. The brown adipocytes of the middle-aged transitional mice appeared “white-like”, and the mitochondrial thermogenesis gene *Ucp-1*, brown tissue marker genes *Cidea* and mitochondrial related genes *Cox7a1* and *Cpt-1m* decreased; the expression of lipolytic genes *Atgl* and *Hsl* were lower relative to adult mice. F72 h significantly reduced the total white BATR (especially mesenteric adipose tissue) of the three groups of mice. Among them, the BATR of various adipose tissue depots of the middle-aged transitional mice showed the smallest decrease; and the expression level of *Atgl* in fat tissues of adult mice increased. After R72 h, the total white BATR of the three groups of mice was still significantly lower compared to the normal feeding group. Among them, the BATR of sWAT returned to normal, while the VAT was still low. The adipose tissue of middle-aged transitional mice reacted slowest to fasting and refeeding cues. Different size and color of adipose tissue represent different morphological changes induced by fasting or refeeding.

The current study used mice with a C57BL/6J background, which have been previously employed as effective models to study obesity and metabolic diseases. This study showed that the bodyweight of mice on a regular chow diet increased significantly between the age of 3 months and 15 months, and a more significant increase in adiposity was observed in the middle-aged transitional mice than their young counterparts, potentially due to adipocyte hypertrophy. Moreover, we observed a slight decrease in ingWAT and a noticeable elevation in both the size and weight percentage of visceral fat with increasing age, particularly the eWAT, which suggested that visceral WAT was a better indicator of aging (Figure 5). In addition, “white-like” morphology (an increase of unilocular adipocytes) was found in iBAT of middle-age transitional mice, indicating morphological impairment. This indicated the impairment of the thermogenic ability of BAT with aging. We also observed that the number of mitochondria was lower in middle-age transitional mice, which plays a major role in mediating thermogenesis in brown adipocytes. The reduced expression of thermogenesis, lipolysis and mitochondrial biogenesis related genes, along with increased lipid biosynthesis genes, indicated a shift in their metabolism towards lipid accumulation rather than oxidation. Thus, aging was found to be associated with a a loss of classical brown adipocytes in mice. Since BAT is responsible for thermogenesis, it resists obesity by increasing energy expenditure, and aging leads to the loss of energy-expending capacity of the BAT, leading to an obesity-prone phenotype in middle-aged mice.

Fasting and subsequent refeeding have been extensively employed to evaluate the effects of various physiological and pathological factors, such as aging and diabetes, on the regulation of metabolism in animals and humans. In our work, we used fasting and refeeding to reveal the aging-associated alterations in lipid metabolism. We found slower metabolic response to fasting in middle-aged transitional mice compared with the young and adult groups. Despite the inconspicuous reduction, the lost body mass and fat mass were not restored after 3 days of refeeding in the middle-aged transitional mice compared to the excessive weight gains in young and adult mice. This indicated a loss of plasticity of adipocytes with aging. Wolden-Hanson and other researchers studied the aging-related regulation of body weight and reported 20% and 14% weight loss after 72 h fasting in adult and middle-aged rats, which was consistent with our results. They also found that aging male rats that failed to increase their food intake after a 72-h fasting and were slow to regain lost body weight on refeeding could be related to an age-related deficiency of NPY mRNA and a blunted fasting-induced increase in NPY gene expression [25]. It has been consistently shown that older animals did not exhibit compensatory hyperphagia as young animals, when challenged with negative energy balance, and their bodyweight decreased or recovered slowly, depending upon the strength of the challenge [26, 27]. Many studies have shown that healthy older people fail to respond to over or under-eating with the compensatory changes in eating observed in younger people [28–31], which may explain the increased susceptibility of older people to chronic conditions like cachexia.

WAT from different anatomical sites at different ages is characterized by different metabolic activities and therefore displays disparate biological functions. Our study showed a considerable reduction in lipid droplet and adipocyte size during fasting, and restoration after refeeding in the three groups. The change was not that significant in middle-aged transitional mice compared with young and adult ones, but the cell size in the middle-aged group was still slightly smaller than that of the fed ones after 72 h refeeding. Analysis of the average area of adipocytes also showed that adipocyte size of mWAT from the three age groups was the first to mobilize. Moreover, the regulation of these processes has been reported to differ greatly between SAT and VAT [28]. In our previous study, we found that fasting preferentially consumed lipids from the visceral white adipose tissue (vWAT) in adult mice [17], and the presence of ‘multilocular’ fat cells mixed with ‘unilocular’ fat cells during fasting in the mWAT implied the browning of white adipocytes. Some studies have demonstrated that calorie restriction promoted browning in WATs [32, 33]. According to our results, such phenomenon disappeared in middle-aged transitional mice, indicating the impaired plasticity of adipose tissue with aging. It has been reported that the various fat depots were differentially affected by aging, which caused the redistribution of fat mass from subcutaneous to visceral locations during aging [34, 35]. Our results showed slower metabolic response to fasting and refeeding in middle-aged transitional mice, indicating an impairment in the plasticity of WAT, which prevented WAT from responding to energy imbalance in time. Age affects the adaptability of adipose tissue to energy changes, making diet control (low-fat and low-calorie diet) very critical for the middle-aged and elderly.

Food deprivation induces lipolysis in adipocytes, while refeeding stimulates lipogenesis. Studies have reported that the activity of lipogenic enzymes in WAT decreases with aging [36]. The delayed recovery of cell size in middle-aged transitional mice refed for 3 days may reflect the reduced lipogenic capacity of adipocytes. Agata Wronska’s work reported the depot-specific, age-related changes in the activities of lipogenic enzymes in white adipocytes [37]. Under fasting conditions, the TGs stored in WAT are hydrolyzed to release free fatty acids (FFAs), which become the primary fuel for liver and muscle through fatty acid oxidation (FAO), called lipid mobilization. ATGL and HSL are the major rate-determining enzymes in this process [38]. Studies showed that the levels of sympathetic nerve-derived catecholamines increased when young (4 months old) and old (21 months old) mice fasted for 24 hours. Fasting increased lipolysis and FFA in young mice, while in the elderly, mice serum FFA decreased, and the body weight and blood glucose did not change significantly, suggesting the decreased lipolytic ability of adipose tissue in elderly mice [39]. Compared with young mice, the release of glycerol and FFA induced by fasting in old mice was significantly reduced and fasting failed to induce the expression of *Hsl* and *Atg1* in old mice [39]. Our analysis of the lipolysis genes provides a plausible interpretation for this reduced lipolytic response in middle-aged mice. In the fed state, the expression of ATGL and HSL were manifold lower in all WAT depots of the middle-aged than in young mice. After 72 h fasting, the expression of both of the above genes were found to be significantly increases in middle-aged transitional mice, which corresponded to the slower reduction in fat mass and cell size. The impaired ability of middle-aged people to mobilize lipids suggests that they should cut back on fats and replace them with complex carbohydrates and proteins in their diets.

Overall, the findings from the present work showed that adipocyte hypertrophy was closely associated with metabolic dysfunction and whiting of BAT, indicating the impairment of the thermogenic ability of BAT in middle-aged transitional mice. This suggested that middle-aged mice were more vulnerable to age-related diseases. We also found that middle-age transitional mice showed delayed metabolic response to fasting and refeeding and speculated that the benefit of dieting was greater and durable in middle-age transitional mice than young mice. However, the damaging effect of age on adipose tissue functioning could be alleviated and was found to be reversible [40]. Understanding the influence of age on adipose tissue characteristics is key to protecting and restoring fat cell function and the physical health of the elderly. In order to prevent and treat obesity in middle-aged people, long-term fasting and refeeding experiments and further human studies are warranted.

## MATERIALS AND METHODS

### Animals

All animal experiments were carried out on juvenile (1-month-old), adult (3-months-old) and middle-aged transitional period (15 months) male C57BL/6J mice that were purchased from the Model Animal Research Center of the Central South University. Animals were housed individually in ventilated Plexiglas cages within a pathogen-free barrier facility and maintained under a 12-h light/12-h dark cycle with a standard rodent chow. For fasting, the mice were housed individually at room temperature (22° C ~ 25° C) with water only. 1-month-old (n =54), 3-months-old (n = 48) and 15-months-old (n = 48) mice were assigned randomly to the following groups: control animals fed ad libitum (Con, n = 5–6), animals fasted for 12 h (F12, n = 5), 24 h (F24, n = 6), 48 h (F48, n = 6) and 72 h (F72, n = 6), respectively, or fasted for 72 h and subsequently refed for 12 h (R12, n = 6), 24 h (R24, n = 6), 48 h (R48, n = 6) and 72 h (R72, n = 6), respectively. Food intake and body weight were measured daily early in the light cycle before the mice were sacrificed.

### Serum and tissue collection

After treatment, mice were anesthetized with isoflurane and blood was taken from the retro-orbital sinus. Blood samples were allowed to clot and were subsequently centrifuged (3500 rpm, 5 min, 4° C). Adipose tissues in the present study were extracted at 9 am every day. Various fat stores, including the main subcutaneous WAT (i.e., ingWAT), the two main visceral WATs (i.e., mWAT and eWAT) and the interscapular BAT (i.e., iBAT) were extracted, weighed, and immediately frozen in liquid nitrogen and stored in -80° C for subsequent RNA isolation and analysis.

### Biochemical assays

Serum concentrations of glucose (GLU), triglyceride (TG), total cholesterol (TC), high density lipoprotein cholesterol (HDL-C), low density lipoprotein cholesterol (LDL-C), non-esterified fatty acid (NEFA), and total protein (TP) were measured in the Department of Laboratory Medicine, The Second Xiangya Hospital, using routine diagnostic tests.

### Hematoxylin and eosin (H&E) staining and adipocyte size measurements

Adipose tissue specimens were individually fixed in 4% paraformaldehyde solution for 24 hours, rinsed with saline, and embedded in paraffin. The samples were stained with H&E for morphological observation. An optical microscope with digital camera (Olympus, Center Valley, PA, USA) was used to collect the images of adipocytes. Three to five images were captured randomly, and then, all images were scanned and counted by the Image-Pro Plus software. Cell areas (μm^2^) were measured and averaged for each section.

### Transmission electron microscopy (TEM)

Transmission electron microscopy investigation was performed using an H-7600 (Hitachi, Japan) at the Electron Microscopy Center of the Central South University. Tissue samples were excised into small pieces (< 1 mm^3^), fixed with 2.5% glutaraldehyde and 1% osmium tetroxide, respectively. Specimens were then dehydrated with graded acetone solutions and embedded in an Epon-Araldite mixture. Thin tissue sections were stained with uranyl acetate and lead citrate. Photomicrographs were taken at 15,000× magnification. The number of mitochondria was analyzed from five to eight randomly delineated micrographs per group using the Image-Pro Plus software.

### Quantitative real-time PCR (qPCR)

Total RNA was extracted from frozen tissue samples using the TRIzol reagent (Thermo Fisher Scientific, MA, USA). Reverse transcription was performed to synthesize cDNA using a RT Kit (Takara, Otsu, Japan). The primers used for real-time qPCR are shown in Table 1. qPCR was performed on a Light Cycler 480 Real time-PCR Detection System (Roche, Basel, Switzerland) using SYBR Green PCR master mix (TaKaRa, Otsu, Japan). Relative mRNA expression was normalized to that of β- actin and expressed as 2^−ΔΔCT^ relative to the control group.

**Table 1:** Real-time PCR primers were used in the present study.

**Gene**	**Forward primer (5′ to 3′)**	**Reverse primer (5′ to 3′)**
*β-actin*	TCGTTACCACAGGCATTGTGAT	TGCTCGAAGTCTAGAGCAAC
*Ucp-1*	AAGCGTACCAAGCTGTGCGA	AGAAAAGAAGCCACAAACCCTT
*Pgc-1α*	TGAACGCACCTTAAGTGTGGAA	GGGTTATCTTGGTTGGCTTTATGA
*Cidea*	ATCACAACTGGCCTGGTTACG	TACTACCCGGTGTCCATTTCT
*Atgl*	AACACCAGCATCCAGTTCAA	GGTTCAGTAGGCCATTCCTC
*Hsl*	TGAGATGGTAACTGTGAGCC	ACTGAGATTGAGGTGCTGTC
*Acox1*	AGGTTGTCATCGCTTTGG	GTGATTAACTCTGGATTGAAGG
*Cpt-1m*	TGCCTTTACATCGTCTCCAA	GGCTCCAGGGTTCAGAAAGT
*Cox7a1*	GTCTCCCAGGCTCTGGTCCG	CTGTACAGGACGTTGTCCATTC
*Hoxc9*	AGACGCTGGAACTGGAGAAGGAG	GCCGCTCGGTGAGATTGAGAAC

### Statistical analyses

Statistical analysis was performed using the SPSS software (version 22.0; IBM, CA, USA) or GraphPad Prism (version 8; GraphPad Software, Inc, San Diego, CA). All values are presented as the mean ± SEM, unless otherwise indicated. Statistical analyses consisted of two-tailed unpaired Student’s t test and one-way ANOVA with post hoc Tukey’s test, with *p* < 0.05 being considered as statistically significant.
